# Supplementary material for: Development of Small Molecule MEIS Inhibitors that modulate HSC activity
Source: Sci Rep. 2020 May 14;10:7994. doi: 10.1038/s41598-020-64888-3 (PMC7224207; doi:10.1038/s41598-020-64888-3)
Supplement: Supplementary file 1 — Supplementary information [file 41598_2020_64888_MOESM1_ESM.pdf]

Supplementary Figures and Files:

Development of Small Molecule MEIS Inhibitors that modulate HSC activity

Raife Dilek Turan[1,2], Esra Albayrak[1], Merve Uslu[1], Pinar Siyah[1], Lamia Yazgi Alyazici[1], Batuhan Mert Kalkan[3], Galip Servet Aslan[4], Dogacan Yucel[5], Merve Aksoz[6], Emre Can Tuysuz[7], Neslihan Meric[1,8], Serdar Durdagi[9], Zafer Gulbas[8], Fatih Kocabas[1,*]

[1]Regenerative Biology Research Laboratory, Department of Genetics and Bioengineering, Faculty of Engineering, Yeditepe University, Istanbul, Turkey
[2]LabCell, Acibadem University, Istanbul, Turkey
[3]Koc University, Istanbul, Turkey
[4]Max Planck Institute for Heart and Lung Research, Germany
[5]Faculty of Medicine, University of Minnesota, Minnesota, USA
[6]MRC Molecular Hematology Unit, Weatherall Institute of Molecular Medicine, University of Oxford, Oxford, UK
[7]Department of Medical Genetics, Faculty of Medicine, Yeditepe University, Istanbul, Turkey
[8]Bone Marrow Transplantation Center, Anadolu Medical Center, Kocaeli, Turkey
[9]Department of Biophysics, School of Medicine, Bahcesehir University, Istanbul, Turkey
*Correspondence:
Fatih Kocabas, Email: Fatih.Kocabas@yeditepe.edu.tr

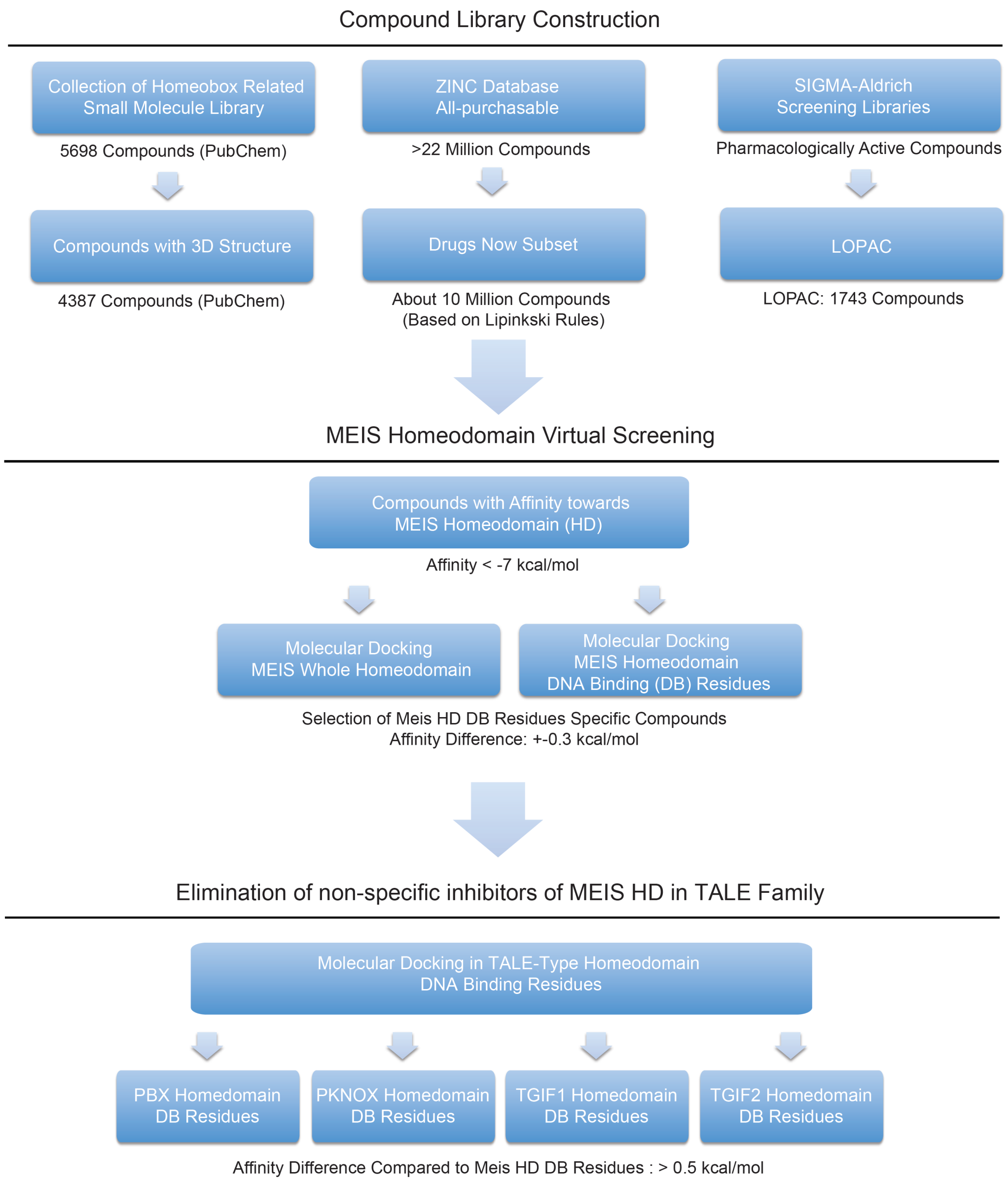

Figure S1 (related to Figure 1). Virtual Screening Strategy. Virtual screening strategy is a tree step process. Initially, *in silico* screening library were generated by compiling SDF files from ZINC drugs-now subset, Sigma LOPAC1280 and curated inhibitors of homeobox family of proteins from PubChem. In the second step, small molecules were docked into whole MEIS homeodomain as well as DNA interacting-highly conserved residues. Final step includes affinity calculation of selected hits against other TALE family proteins to eliminate non-specific hits.

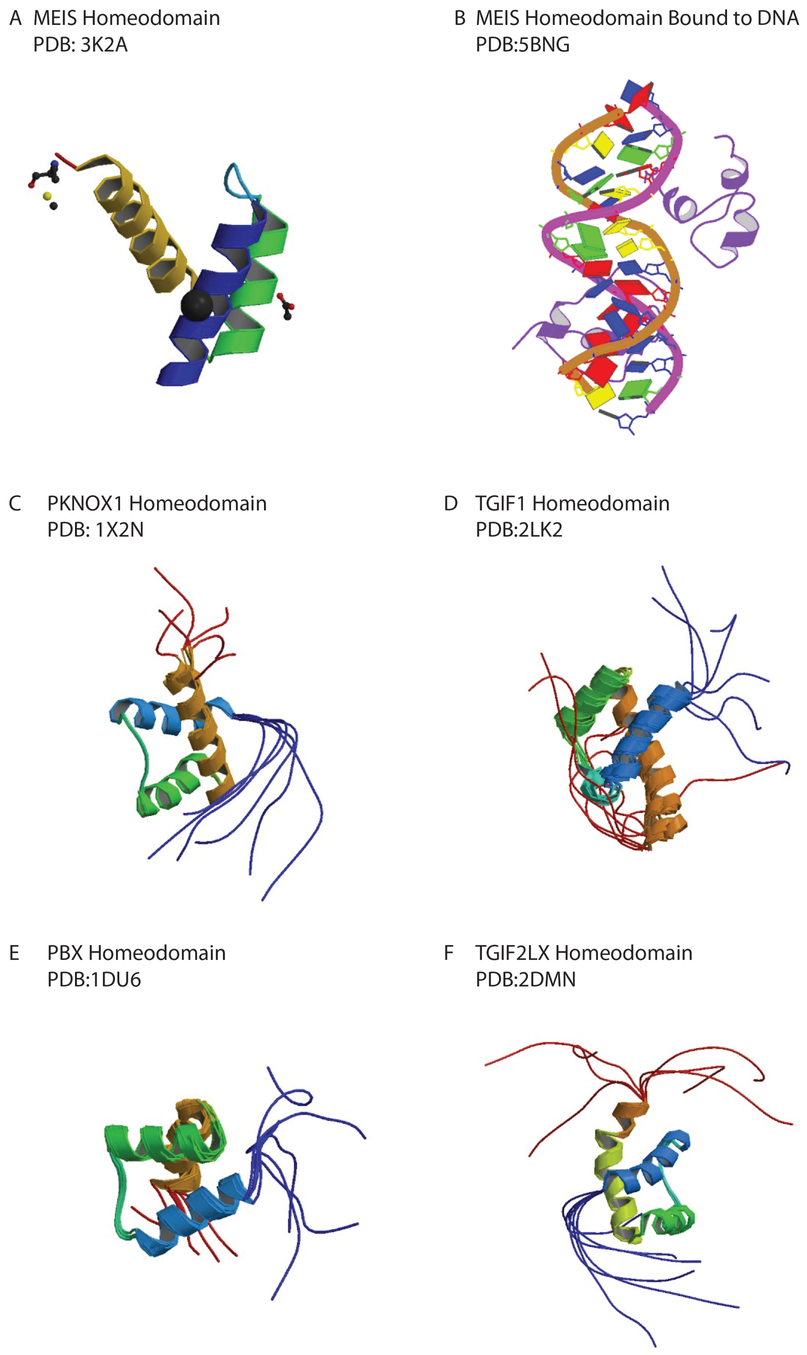

Figure S2 (related to the Figure 1). Crystal Structure of TALE-type homeodomains. TALE family proteins with known homeodomain crystal structure have been obtained from PDB and docked with MEIS small molecule hits. Gibbon diagrams for **A)** MEIS HD, **B)** MEIS HD with DNA, **C)** PKNOX HD, **D)** TGIF1 HD, **E)** PBX HD, **F)** TGIF2LX HD are shown. Grid boxes for docking were generated around DNA binding helices.

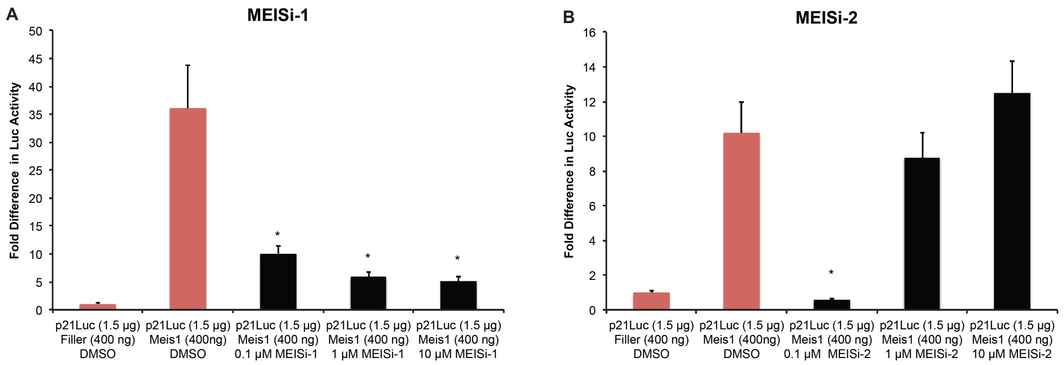

Figure S3 (related to Figure 2). Dose dependent effect of MEISi-1 and MEISi-2 in MEIS luciferase reporter activity. Luciferase reporter assays demonstrate the dose dependent effect of **A)** MEISi-1 and **B)** MEISi-2 compared to DMSO control. n=3, *$p<0.05$.

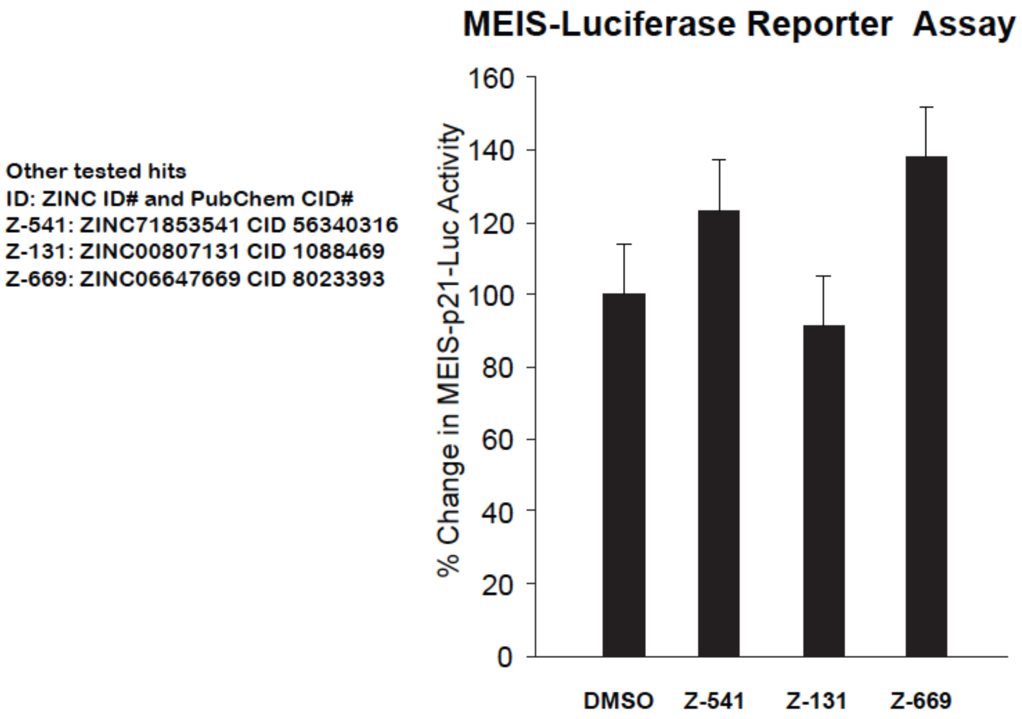

Figure S4 (related to Figure 2). Other tested MEIS hits with similar structure in MEIS luciferase reporter assay.

Figure S5 (related to Figure 2). MEIS inhibitors tested in PBX-Luc Reporter

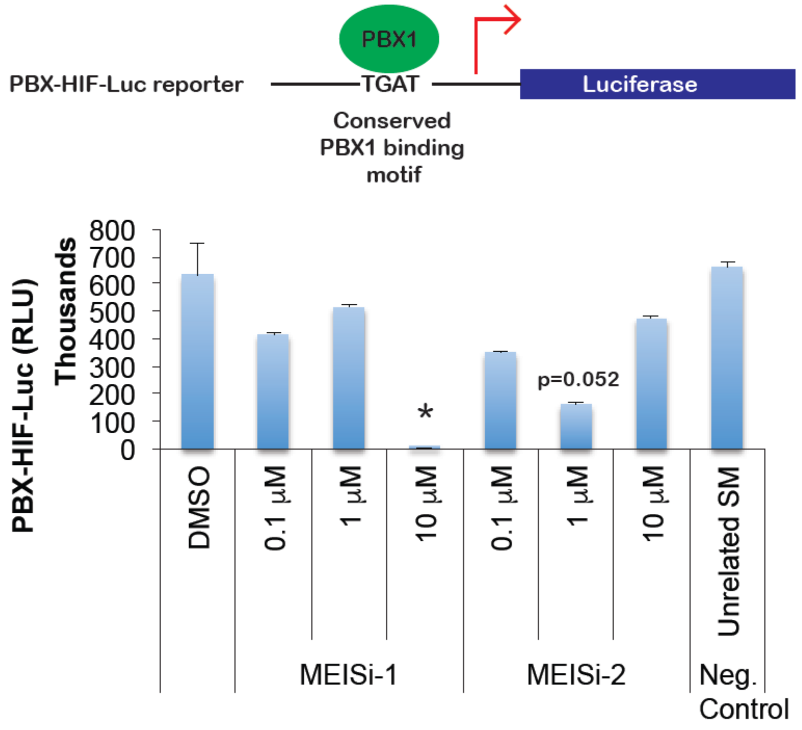

Figure S5 (related to Figure 2). MEIS inhibitors tested in PBX-Luc Reporter. Analysis of other MEISi-1 and MEISi-2 in PBX-luc-reporter assay. n=3, *p<0.05.

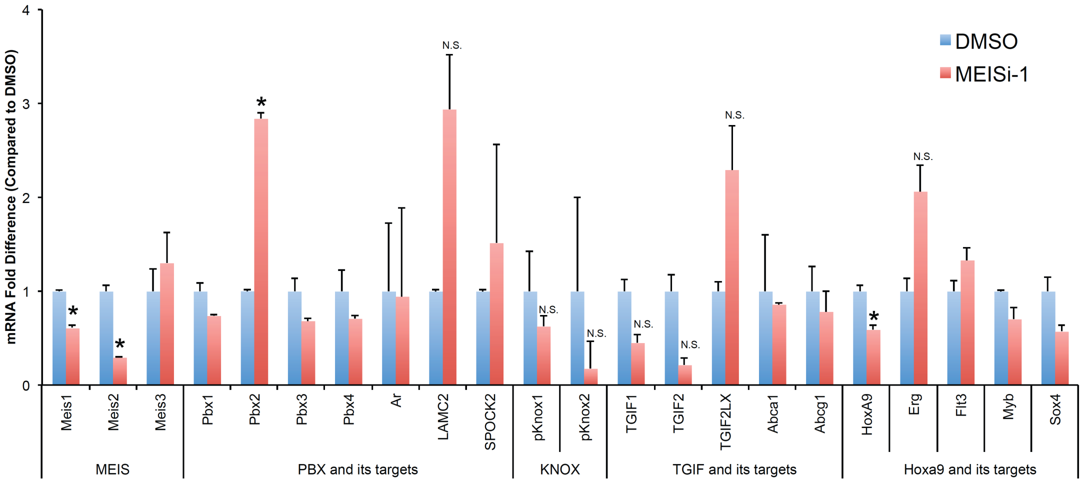

Figure S6 (related to Figure 2). Gene expression analysis of TALE family genes and their targets. We have determined gene expression analysis post MEISi-1 to determine how MEIS inhibitors affect expression of Meis1, Meis1 related, other TALE family genes, and more importantly their target genes. n=3, *p<0.05, N.S.=Not significant.

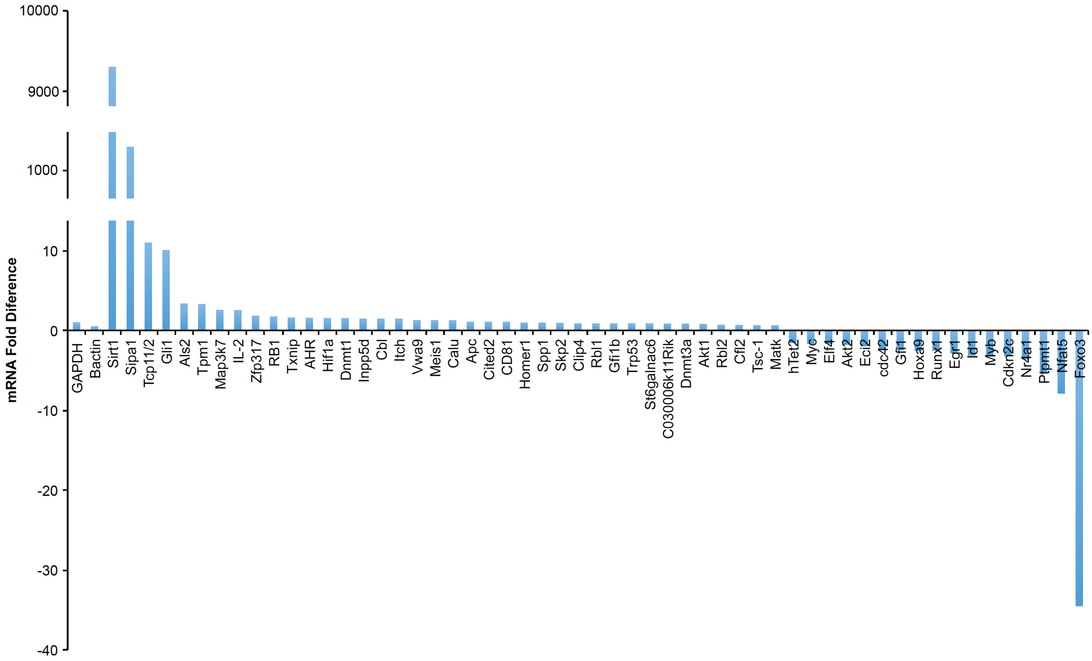

Figure S7 (related to Figure 2). Analysis of HSC gene pool by PCR array post MEISi-1 treatment in Lin- Cells. We have determined gene expression analysis post MEISi-1 to determine how MEIS inhibitors affect expression of HSC related genes.

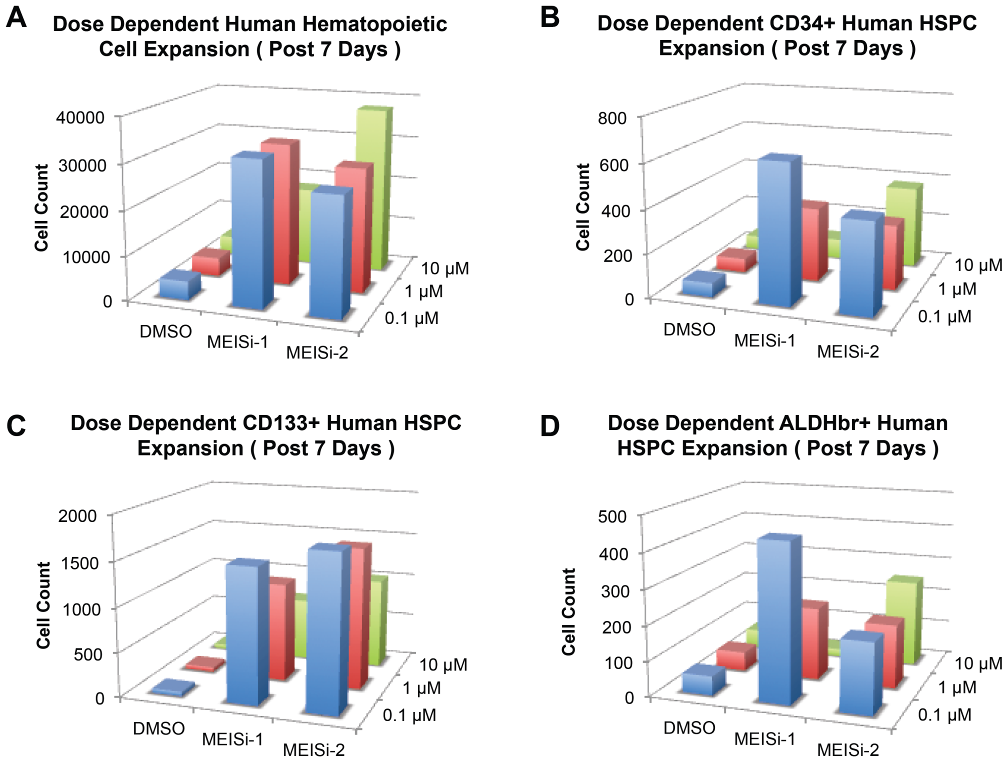

Figure S8 (related to Figure 4). Human UCB hematopoietic cell expansion post MEISi treatments. Human UCB MNCs were treated with three different doses of MEISi-1 and MEISi-2. **A)** Human hematopoietic cell count, **B)** CD34^{+} human HSPC, **C)** CD133^{+} human HSPC count, **D)** ALDH^{br} human HSPC counts were determined at given concantrations.

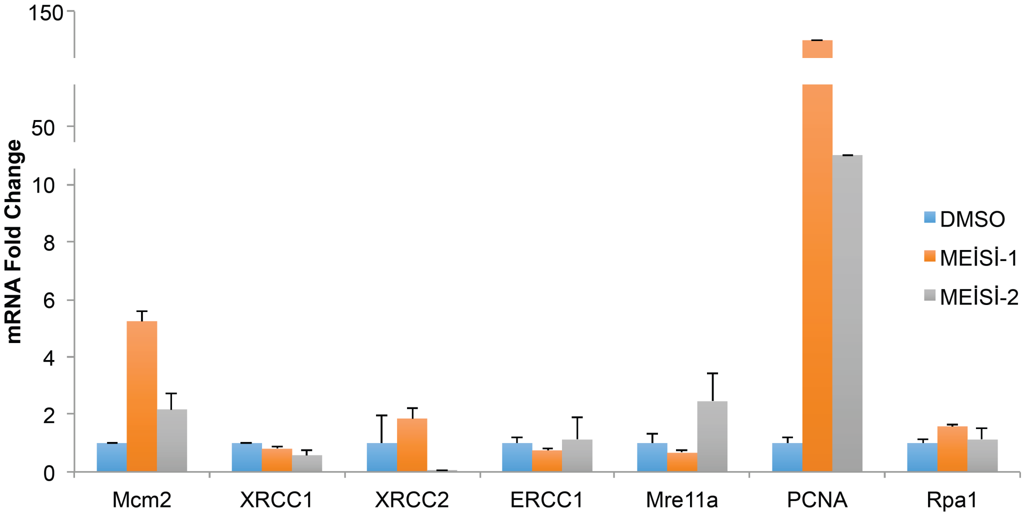

Figure S9. Effect of MEISi treatments in the expression of HDR related genes. Lin- cells were treated *in vitro* with MEIS inhibitors and collected RNA post 3 days of treatment for analysis of gene expression. n=3.

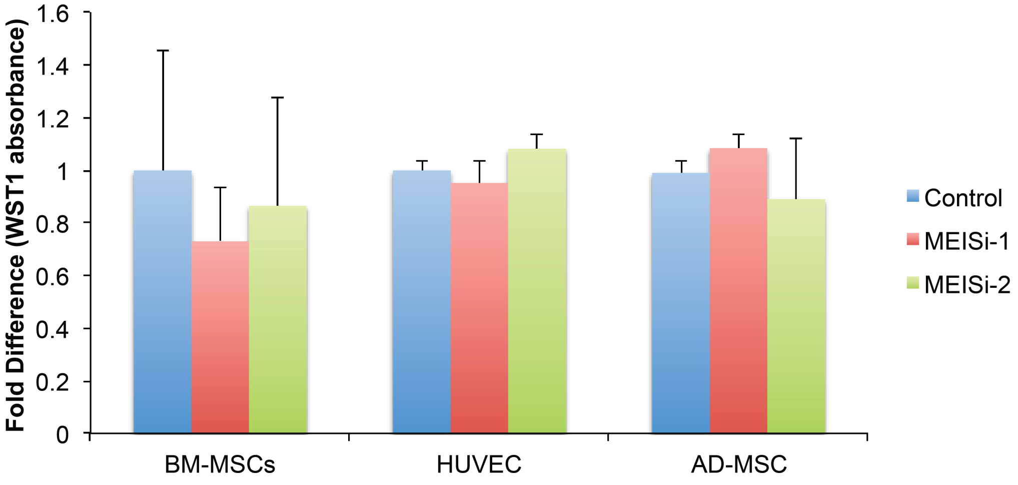

Figure S10. Effect of MEISi treatments in BM-MSC, HUVEC and AD-MSC proliferation post 4 days. BM-MSCs, HUVECs, and AD-MSCs were treated with 1 μM of MEISi-1, MEISi-2 inhibitors and DMSO (control, 0.5%). Fold difference in WST1 absorbance was determined after 72 hours. n=3.

Supplementary Files

Supplementary File 1 (related to Figure 1): Enrichment analysis.

Supplementary File 2 (related to Figure 1 and Table 5): Selected hit small molecules with no predicted cardiotoxicity. Hit molecules were docked with Glide/XP into hERG. Affinities predicted to be <-7.8 kcal/mol (Maestro Glide) or <-7.4 kcal/mol (AutoDockVina) are considered potentially cardiotoxic.

Data availability: Additional data available upon request.
